# Case Report: IgG4-related autoimmune pancreatitis presenting as an infiltrative cystic-solid pancreatic mass: lessons from a diagnostic pitfall

**DOI:** 10.3389/fmed.2026.1756102

**Published:** 2026-04-02

**Authors:** Ran Xiao, Tianyu Li, Bo Chen, Liang Zhu, Liangbo Dong, Yaozong Wang, Xiaoyuan Qiu, Jingyi Liu, Mingliang Bai, Jiashu Han, Chen Lin, Weibin Wang

**Affiliations:** 1 Department of Radiology, Peking Union Medical College Hospital, Peking Union Medical College, Chinese Academy of Medical Sciences, Beijing, China; 2 Department of General Surgery, Peking Union Medical College Hospital, Peking Union Medical College, Chinese Academy of Medical Sciences, Beijing, China; 3 Department of Pathology, Peking Union Medical College Hospital, Peking Union Medical College, Chinese Academy of Medical Sciences, Beijing, China; 4 Department of Surgery, Zhouqu County People’s Hospital, Longnan, Gansu, China

**Keywords:** dense fibrosis, diagnostic pitfall, FDG-PET/CT, histopathology, IgG4-related autoimmune pancreatitis, infiltrative cystic-solid pancreatic mass, invasive pancreatic carcinoma, lymphoplasmacytic infiltration

## Abstract

IgG4-related autoimmune pancreatitis (AIP) can resemble pancreatic ductal adenocarcinoma (PDAC), but it typically presents as a solid mass rather than a cystic-solid lesion. We report a rare case of AIP in a middle-aged man with a long-standing pancreatic tail mass that gradually enlarged and developed a cystic-solid configuration. Photon-counting CT showed a non-enhancing cystic component and a progressively enhancing solid portion with apparent invasion of adjacent organs, while FDG-PET/CT demonstrated marked metabolic activity, all strongly suggestive of malignancy. Serum IgG4 levels were normal and no extrapancreatic IgG4-related involvement was present. The patient declined biopsy and underwent radical surgery; histopathology revealed dense fibrosis, pancreatic atrophy, and lymphoplasmacytic infiltration consistent with IgG4-related AIP, with no evidence of cancer. This case highlights that atypical cystic-solid AIP can closely mimic invasive PDAC even with advanced imaging techniques, underscoring the importance of recognizing such rare presentations to avoid unnecessary radical resection.

## Introduction

IgG4-related disease is a fibro-inflammatory condition that can involve nearly all organ systems (1). Common manifestations include autoimmune pancreatitis, orbital disease, tubulointerstitial nephritis, and retroperitoneal fibrosis. Its characteristic histopathological features include dense, polyclonal lymphoplasmacytic infiltration enriched with IgG4-positive plasma cells (with an IgG4/IgG ratio >40%), obliterative phlebitis, and storiform fibrosis (2).

Type 1 AIP essentially represents the pancreatic manifestation of IgG4-related disease. It is driven by a Th2/Treg-dominant immune response and often mimics pancreatic cancer in both clinical and imaging presentations (3), yet it responds well to corticosteroids, has a high relapse rate, and may progress to chronic pancreatitis over time (4). While focal AIP most often manifests as a solid, delayed-enhancing mass, cystic-solid configurations are exceedingly uncommon and may further obscure the diagnosis. Advanced imaging techniques and multimodal approaches have considerably enhanced lesion characterization in the differential diagnosis between AIP and pancreatic cancer. However, atypical manifestations may still mimic malignancy and lead to misinterpretation. Moreover, metabolic imaging offers limited specificity in this setting, as active inflammatory lesions in AIP can exhibit markedly increased FDG uptake, sometimes indistinguishable from malignant tumors.

Cystic or cystic-solid presentation of autoimmune pancreatitis (AIP) is rare, accounting for fewer than 5% of cases. Reports of invasive-appearing cystic-solid AIP are limited to isolated cases. This atypical morphology substantially increases the risk of misdiagnosis as PADC, highlighting the clinical relevance of the present case. Here, we present a highly unusual case of AIP manifesting as a large cystic-solid mass with an infiltrative appearance on photon-counting CT and intense FDG uptake, leading to a preoperative diagnosis of invasive pancreatic carcinoma.

## Case presentation

A 43-year-old man was referred for evaluation of a pancreatic tail mass that had been incidentally detected several years earlier. The lesion enlarged progressively during follow-up, accompanied by a gradual rise in CA19-9 levels. The serum CA19-9 increased from 63.7 U/mL at initial presentation to 214.2 U/mL preoperatively. The patient reported persistent left upper abdominal discomfort radiating to the back but had no history of acute pancreatitis attacks or chronic pancreatitis, and no evidence of systemic IgG4-related disease. Physical examination was unremarkable except for mild left flank tenderness. Serum IgG4 levels remained within the normal range.

**Figure 1:**
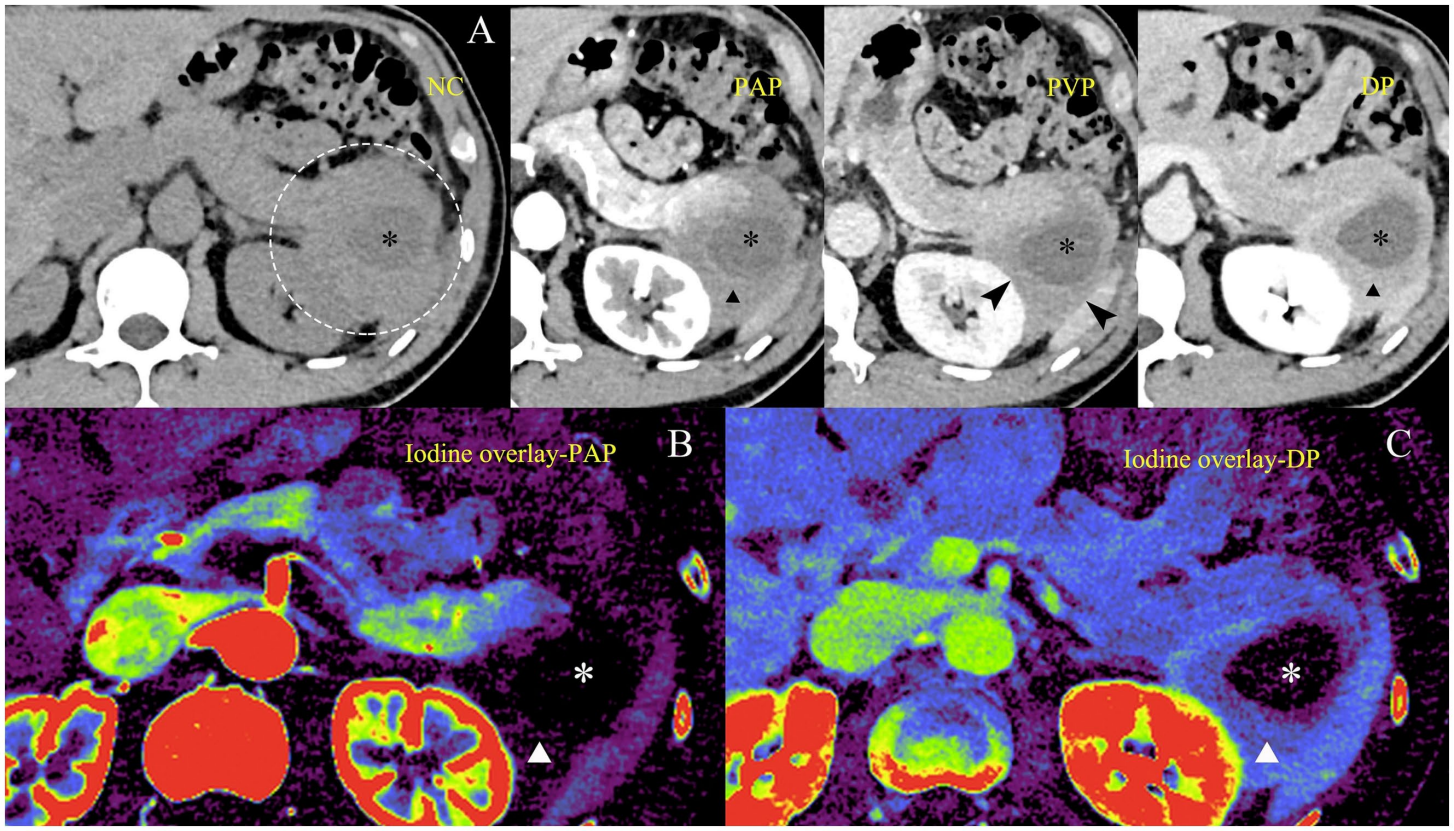
Multiphasic CT and iodine map images of the pancreatic lesion. **(A)** Axial contrast-enhanced CT demonstrates a cystic-solid lesion infiltrating the pancreatic tail (dotted circle). The cystic component (black asterisk) shows no enhancement, while the solid portion (black triangle) exhibits mild enhancement on the pancreatic parenchymal phase (PAP) and progressive enhancement on the delayed phase (DP). The lesion abuts the left kidney and spleen [black arrowheads, portal venous phase (PVP)] with loss of the intervening fat plane but without definite invasion. No typical IgG4-related changes are observed in the kidneys, aorta, or other abdominal organs. **(B,C)** Iodine maps on PAP and DP show mild iodine uptake in the solid component (white triangle) and absence of measurable iodine in the cystic portion (white asterisk). The gradual increase in iodine concentration in the solid component during DP parallels its progressive enhancement, suggesting fibrous stroma or low vascularity. NC, non-contrast; PAP, pancreatic parenchymal phase; PVP, portal venous phase; DP, delayed phase.

Photon-counting CT revealed a large cystic-solid mass measuring approximately 7 cm in the pancreatic tail. The cystic component showed no iodine uptake, while the solid portion exhibited mild enhancement in the pancreatic phase and progressive enhancement in the delayed phase. The lesion abutted the spleen and left kidney, with loss of the intervening fat plane, suggesting potential invasion (Figure 1A). No typical IgG4-related changes were identified in the kidneys or biliary system. Iodine map analysis revealed mild iodine uptake in the solid portion and no measurable iodine in the cystic component during the pancreatic parenchymal phase; in the delayed phase, the iodine concentration in the solid portion further increased (Figures 1B,C). FDG-PET/CT demonstrated marked metabolic activity within the solid component (SUV_max_ 8.3).

**Figure 2:**
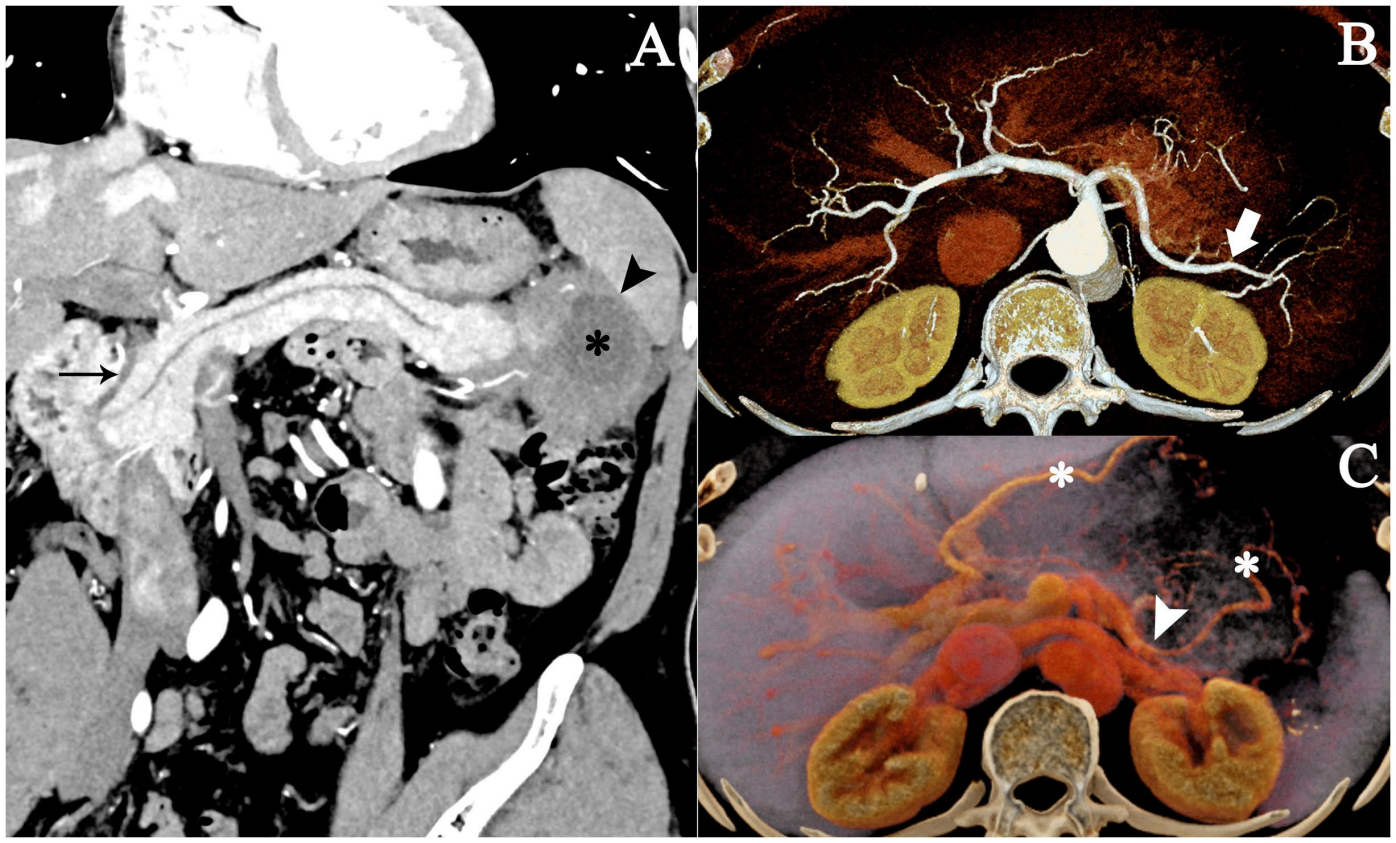
Curved planar and volume-rendered reconstructions from contrast-enhanced CT. **(A)** Curved planar reconstruction (PAP, 1 mm slices) reveals a cystic-solid lesion in the pancreatic tail (black asterisk) with an irregular contour abutting the spleen (black arrowhead) and obscuration of the fat plane. The common bile duct appears slender and smooth (thin black arrow), without typical features of IgG4-related disease. **(B,C)** Cine volume-rendered reconstructions (0.4 mm PAP and PVP images) clearly delineate the celiac trunk and major arterial branches, as well as the portal venous system. The lesion encases the distal splenic artery (solid white arrow) and splenic vein (white arrowhead). The distal splenic artery and its branches exhibit mild luminal narrowing, while the splenic vein gradually tapers toward the hilum to complete occlusion, accompanied by multiple collateral veins (white asterisks). PAP, pancreatic parenchymal phase; PVP, portal venous phase.

Curved planar reconstruction images showed an irregularly shaped lesion adjacent to the spleen with loss of the fat plane; the common bile duct was slender and smooth, without typical IgG4-related changes (Figure 2A). Dynamic volume-rendered reconstruction images clearly visualized the celiac trunk and its major branches, as well as the portal venous system. The lesion encased the distal splenic artery and splenic vein; the distal splenic artery and its branches showed mild luminal narrowing, while the splenic vein tapered gradually toward the hilum and became occluded, with multiple collateral veins visible (Figures 2B,C). Due to the patient’s refusal of biopsy, surgery was decided after multidisciplinary consultation.

The patient underwent robot-assisted distal pancreatectomy with splenectomy and partial colectomy. Intraoperatively, a firm 8 cm mass was observed in the pancreatic tail, densely adherent to the splenic flexure and renal fascia. Frozen section revealed chronic pancreatitis with arteritis, suggesting a possible autoimmune etiology. Permanent pathological examination confirmed extensive fibrosis, pancreatic atrophy, and marked lymphoplasmacytic infiltration with lymphoid follicle formation, but no evidence of malignancy. Immunohistochemical results showed: anaplastic lymphoma kinase (ALK) (−), cytokeratin 7 (CK7) (−), CD20 (+), CD3 (+), CD38 (+), CD138 (focal +), multiple myeloma oncogene 1 (MUM-1) (+), smooth muscle actin (SMA) (−), and Ki-67 positivity of 70% in germinal centers. The IgG4/IgG ratio <40%, with approximately 35 IgG4-positive plasma cells per high-power field. Representative histopathological and immunohistochemical images are provided in Supplementary Figure 1. Postoperatively, the patient developed a transient pancreatic fistula with elevated drain amylase (48,000 U/L) and *Pseudomonas* infection, which resolved with meropenem and metronidazole. The patient recovered uneventfully and was discharged on postoperative day 18. At the 3-month follow-up, CA19-9 normalized, and magnetic resonance imaging (MRI) showed no signs of recurrence.

## Discussion

This case highlights the diagnostic challenges of differentiating IgG4-related autoimmune pancreatitis from pancreatic carcinoma, even with state-of-the-art imaging techniques. Typical imaging findings of IgG4-related AIP follow a relatively characteristic pattern, including diffuse “sausage-like” enlargement of the pancreas, homogeneous delayed enhancement, a capsule-like rim, and long-segment narrowing of the main pancreatic duct with only mild upstream dilation (4, 5). These classic features allow AIP to be distinguished from PDAC in many cases. However, focal AIP is far more heterogeneous and may closely mimic malignant tumors, particularly when the hallmark capsule-like rim or extrapancreatic IgG4-related changes are absent.

In contrast to these typical patterns, our present case exhibited markedly atypical imaging characteristics, most notably a prominent cystic-solid configuration, in which the cystic component showed no iodine uptake while the solid portion demonstrated progressive delayed enhancement. The cystic component likely reflects secondary changes from chronic fibro-inflammatory injury rather than tumor necrosis. In AIP, long-standing inflammation may cause ductal obstruction and parenchymal atrophy, leading to pseudocyst formation or degenerative cystic change. This differs from PDAC, where cystic degeneration typically results from rapid tumor growth and central necrosis.

The lesion also displayed extensive contact with adjacent organs and loss of intervening fat planes, creating a distinctly infiltrative appearance that strongly resembled invasive PDAC. Such cystic-solid morphology is exceedingly rare in AIP, which more commonly presents as a homogeneous or mildly heterogeneous solid mass. Although PET/CT showed markedly increased FDG uptake in the solid component, this finding does not reliably distinguish malignancy from AIP, as active IgG4-related inflammation can produce equally intense or even higher FDG activity than pancreatic cancer (6–8).

Another notable feature in this case is the presence of an irregular thick wall with gradual enhancement surrounding the non-enhancing cystic component. This morphology may represent a variant of the “capsule-like rim” characteristic of IgG4-related AIP. Histologically, the typical capsule corresponds to dense peripancreatic inflammatory fibrosis, appearing as a low-attenuation halo on CT and a T2-hypointense rim on MRI (8, 9). In atypical or long-standing lesions, this inflammatory rim may become irregular and focally thickened. The infiltrative thick-wall of the cystic-solid lesion, initially considered a sign of malignancy, is therefore more likely attributable to localized peripancreatic fibrosing inflammation.

Histologically, classic type 1 AIP is characterized by dense lymphoplasmacytic infiltration, storiform fibrosis, and obliterative phlebitis, along with elevated IgG4-positive plasma cells (10, 11). In this case, the lesion lacked storiform fibrosis and had an IgG4/IgG nearly reach the typical diagnostic threshold, representing a localized or late-stage “burnt-out” lesion. In focal disease, inflammation may remain confined to the pancreas without systemic immune activation. The coexistence of pseudocystic change, absence of extrapancreatic IgG4 involvement, and normal serum IgG4 further contributed to diagnostic ambiguity. These atypical or partial forms reinforce the need to consider AIP in the differential diagnosis of any pancreatic mass, even when serology is negative.

Previous reports have emphasized that AIP and PDAC share overlapping imaging characteristics (12, 13). Despite improved resolution with photon-counting CT, distinguishing between inflammatory and malignant lesions remains problematic. Recognition of systemic IgG4-related manifestations and histopathological confirmation are critical for accurate diagnosis. Clinically, several key features of this case warrant emphasis to refine diagnostic reasoning. The lesion’s six-year indolent course, from initial detection as a 2 cm asymptomatic lesion to gradual enlargement without acute deterioration, differs from the more aggressive progression typically seen in pancreatic cancer (14). Additionally, the moderate and stepwise elevation of CA19-9 is inconsistent with the often rapid and marked CA19-9 rise in advanced malignant disease (15). It is also critical to recognize that FDG avidity on PET/CT is a non-specific finding. While high SUV_max_ values is frequently associated with malignancy, robust inflammatory responses in fibro-inflammatory disorders like AIP can induce comparable metabolic activity via increased immune cell proliferation and cytokine release (16, 17). These clinical and metabolic clues, when integrated with imaging findings, should prompt a higher index of suspicion for inflammatory etiologies and underscore the importance of pursuing histopathological confirmation. Whenever technically feasible, image-guided core needle biopsy should be prioritized before proceeding with radical surgical interventions, as it can provide definitive tissue diagnosis to distinguish between AIP and carcinoma.

To reduce unnecessary radical surgery when AIP mimics PDAC, a guideline-based diagnostic strategy is crucial. Imaging should be interpreted alongside ductal evaluation, as fibro-inflammatory changes may simulate invasive malignancy. Serum IgG4 testing and assessment for extra-pancreatic involvement are recommended, recognizing that seronegativity does not exclude type 1 AIP. When feasible, EUS-guided core biopsy should be prioritized for histologic confirmation. In carefully selected cases with reasonable exclusion of cancer, a monitored steroid trial may aid diagnosis-radiologic response supports AIP, whereas lack of response raises concern for malignancy. Multidisciplinary evaluation is essential to integrate clinical, imaging, and pathologic findings and avoid overtreatment.

In conclusion, this case demonstrates that even photon-counting CT cannot always distinguish atypical IgG4-related autoimmune pancreatitis from invasive pancreatic carcinoma. Awareness of such imaging pitfalls, careful clinical correlation, and multidisciplinary collaboration are vital to avoid unnecessary radical surgery.

## Data Availability

The original contributions presented in the study are included in the article/Supplementary material, further inquiries can be directed to the corresponding authors.
