# Supplementary figures and images for: Case Report: IgG4-related autoimmune pancreatitis presenting as an infiltrative cystic-solid pancreatic mass: lessons from a diagnostic pitfall
Source: Front Med (Lausanne). 2026 Apr 2;13:1756102. doi: 10.3389/fmed.2026.1756102 (PMC13082946; doi:10.3389/fmed.2026.1756102)

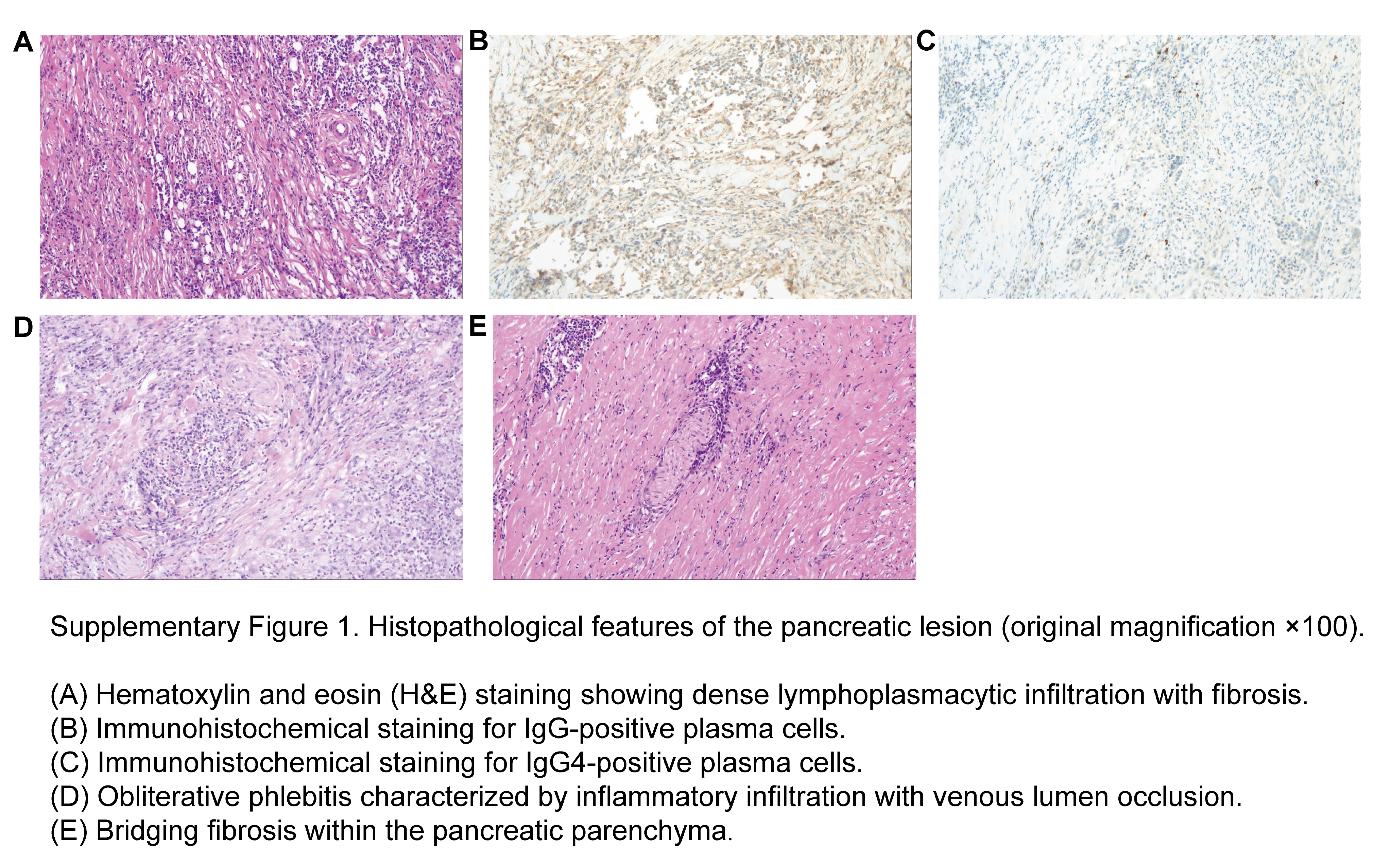

Supplement: Supplementary file 1 [file Image_1.jpeg]
